# Application of a Novel “Pan-Genome”-Based Strategy for Assigning RNAseq Transcript Reads to *Staphylococcus aureus* Strains

**DOI:** 10.1371/journal.pone.0145861

**Published:** 2015-12-30

**Authors:** Diego Chaves-Moreno, Melissa L. Wos-Oxley, Ruy Jáuregui, Eva Medina, Andrew P. A. Oxley, Dietmar H. Pieper

**Affiliations:** 1 Microbial Interactions and Processes Research Group, Helmholtz Centre for Infection Research, Braunschweig, Germany; 2 Infection and Immunology Research Group, Helmholtz Centre for Infection Research, Braunschweig, Germany; University of North Carolina at Charlotte, UNITED STATES

## Abstract

Understanding the behaviour of opportunistic pathogens such as *Staphylococcus aureus* in their natural human niche holds great medical interest. With the development of sensitive molecular methods and deep-sequencing technology, it is now possible to robustly assess the global transcriptome of bacterial species in their human habitat. However, as the genomes of the colonizing strains are often not available compiling the pan-genome for the species of interest may provide an effective method to reliably and rapidly compile the transcriptome of a bacterial species. The pan-genome of *S*. *aureus* and its associated core and accessory components were compiled based on 25 genomes and comprises a total of 65,557 proteins clustering into 4,198 Orthologous Groups (OGs). The generated gene catalogue was used to assign RNAseq-derived sequence reads to *S*. *aureus* in a variety of *in vitro* and *in vivo* samples. In all cases, the number of reads that could be assigned to *S*. *aureus* was greater using the OG database than using a reference genome. Growth of two *S*. *aureus* strains in synthetic nasal medium confirmed that both strains experienced strong iron starvation. Traits such as purine metabolism appeared to be more affected in a typical nasal colonizer than in a strain representative of the *S*. *aureus* USA300 lineage. Mapping sequencing reads from a metatranscriptome generated from the human anterior nares allowed the identification of genes highly expressed by *S*. *aureus in vivo*. The OG database generated in this study represents a useful tool to obtain a snapshot of the functional attributes of *S*. *aureus* under different *in vitro* and *in vivo* conditions. The approach proved to be advantageous to assign sequencing reads to bacterial strains when RNAseq data is derived from samples where strain information and/or the corresponding genome/s are unavailable.

## Introduction

RNAseq is an approach that uses deep-sequencing technology to generate a high-resolution transcriptome map of a genome or a community meta-genome under a specific condition in time. The principle of RNAseq is to generate sequence reads from random locations along the individual RNA molecules within the total RNA pool [1]. To obtain a detailed view of the transcriptional profile of different bacterial community members, the sequenced short-reads are then mapped against a reference genome or catalogue of sequences such as a meta-genome library. However, this method is unsuitable for samples lacking precise reference genomes and/or sequence catalogues. However, with the recent advent of whole-genome sequencing on a massive scale [2], a large number of genomes are now available, with >2,800 finished bacterial genomes currently listed in the Integrated Microbial Genomes system (https://img.jgi.doe.gov). All this information enables the gathering and compilation of genes of a given species into unique databases that can then be used for assigning RNAseq transcripts as alternative to the use of a reference genome.

The entirety of a species gene repertoire, the “pan-genome” comprises a core of genes shared by all strains within a given species, and a dispensable or accessory genome composed of genes that are only present in some strains or that are strain-specific [3,4]. Differences in the pan-genome are reflections of the lifestyle and evolutionary history of an organism and the pan-genome may be defined as being open or closed. As originally demonstrated for *Streptococcus agalactiae* [3] and *Haemophilus influenzae* [5], the pan-genome is suitable for ascribing a bacterial species. Thus it can be used for assigning genes to strains belonging to the same species for which the genomes are uncharacterised. This has become particularly relevant for assigning genes to individual bacterial strains originating from complex communities. The human anterior nares provide a good example since they are inhabited by commensals and often also by a suite of important opportunistic pathogens [6] such as *Staphylococcus aureus*. Although RNAseq is an excellent tool for assessing the behaviour of *S*. *aureus* in its natural niche, the precise reference genomes may not be available for the specific colonizing strains. Thus, an alternative approach for optimal assignment of transcripts must be undertaken, specifically as staphylococcal carriage can be highly dynamic with the concomitant acquisition and loss of different strains over the short and long-term [7]. In this regard, the pan-genome can constitute an attractive reference tool for assigning transcripts to specific organisms in complex metatranscriptomes. This method could be particularly suitable for those organisms like *S*. *aureus*, which have been described as having seemingly bounded (or closed) genomes and where the majority of the gene content may be captured by a relatively small number of genome sequences [4,8,9].

The aim of the current study was to compile and assess the “pan-genome” of *S*. *aureus* and to create an *S*. *aureus* gene catalogue for assigning transcription reads derived from RNAseq analysis. This catalogue is based on Orthologous Groups or (OGs) of genes encoding proteins which share a common ancestor and probably biochemical function defined for the species of interest. The suitability of this method was tested in divergent *S*. *aureus* strains growing under different conditions in *vitro* as well as in a complex metatranscriptome sample from a colonized individual and was compared with the method of assigning reads using a reference genome.

## Materials and Methods

### Retrieval of genome sequences and metadata

The finalized genome sequences of a total of 32 *S*. *aureus* strains were retrieved from the National Center for Biotechnology Information (NCBI) ftp site (S1 Table) as well as the corresponding protein, nucleotide and annotation (GFF and PTT) files used for comparative analyses and establishing the “pan-genome” of *S*. *aureus*.

### Orthologous group search, PSSM database construction and COG assignment

As a first step in establishing the pan-genome, the minimum number of strains required to yield a relatively invariable genome content was assessed by a nested comparison of Orthologous Groups (OGs) between all 32 genomes. The genomes were sorted in ascending order according to the number of proteins and the OGs identified through an all-against-all protein sequence comparison using blastp [10], followed by Markov clustering (MCL) with an inflation factor of 1.5 as described in OrthoMCL [11]. Genomes were compared in an iterative and additive manner; beginning with a comparison between the 2 genomes with the lowest protein counts. This group was subsequently compared to the next genome comprising the next lowest protein counts and the process repeated a total of 31 times adding one genome per iteration. The total numbers of unique and shared OGs comprised within the genomes per iteration was calculated and plotted as saturation (and fit to power) curves, representing the pan- and core-genomes respectively. A sub-set of 25 genomes, corresponding to a point of relative stability in the OG content, was used as a final group of representative genomes. An OG search was further conducted on this group of 25 genomes (comprising a total of 65,557 proteins) using the same methodology, yielding a total of 4,198 OGs. To evaluate the variation between these strains based on OG content, the number of proteins was calculated for each OG per genome, tabulated and agglomerative hierarchical cluster analysis applying the Bray-curtis similarity algorithm (using PRIMER-E software [12]) was performed.

The corresponding protein sequences for each of the 3,466 core and accessory OGs were subsequently retrieved and aligned with MUSCLE [13] (using default parameters) and each alignment converted into a Position Specific Scoring Matrix (PSSM) database using the psi-blast program contained as part of the blast suite version 2.2.25 [10] and formatted using the associated Formatrpsdb script. In addition, a consensus sequence was generated for each of the alignments using the cons program of the EMBOSS suite version 6.3.1 [14]. These PSSM databases were used in the downstream interrogation of *S*. *aureus* transcript reads generated from the RNAseq libraries (see below). The database was re-built using the genomes of *S*. *aureus* EMRSA16 and *S*. *aureus* HO 5096 0412 retrieved from the NCBI ftp site. Orthologous groups were identified as explained above.

### Calling metatranscriptomic reads afiliated with *Staphylococcus* spp.

In order to determine the best approach for identifying sequence reads specific to *S*. *aureus* from complex *in vivo* samples where cohabitation of closely related species (namely *S*.*epidermidis*) is typical, the complete genome sequences of *S*. *aureus* 6850, *S*. *epidermidis* RP62A and *D*. *pigrum* ATCC 51524 were retrieved from the NCBI ftp website. A total of 6000 reads (of 100 nt in length) were randomly extracted in different proportions into one of three files using an in-house Ruby script. The proportions of reads assigned to each file were predefined to comprise equal quantities (33%) of each genome in file 1, 10% *S*. *aureus* and 90% *S*. *epidermidis* in file 2, and 70% *S*. *aureus*, 20% *S*. *epidermidis* and 10% *D*. *pigrum* in file 3.

Each file of random sequences was evaluated by blast searches against a nucleotide sequence database comprising the complete genomes from the 25 *S*. *aureus* strains used here (S1 Table) and a database consisting of the sequences from the *S*. *epidermidis* strains ATCC 12228 and RP62A. An alignment score considering the % identity and the % coverage of the query (alignment length × % identity/query length) was calculated for each blast search and alignment scores > of 70% and > 80% used for the assignment of reads as corresponding to either *S*. *aureus*, *S*. *epidermidis* or *D*. *pigrum*.

### Strains and *in vitro* cultures

For testing the applicability of a pan-genome approach for assigning transcriptomic (RNAseq) reads from individual strains, 2 strains of *S*. *aureus* were used, *S*. *aureus* USA300 strain LAC [15] (USA300-0114, National Institutes of Health, BEI Resources catalog number NR-46070) representing the USA300 lineage (multilocus sequence type 8, clonal complex 8) where finished genome sequence of that lineage are available and *S*. *aureus* IPL32 [16], a human nasal MRSA isolate from Northern Germany belonging to the Barnim strain group (EMRSA-15, sequence type 22, clonal complex 22, spa-type t032, the predominant MRSA lineage in Europe). Both strains were grown in 500 ml erlenmeyer flasks containing 100 ml of Brain Heart Infusion (BHI) media (Roth; Karlsruhe, Germany) or 100 ml of Synthetic Nasal Medium (SNM) developed to represent the secretions of the human nares [17] with shaking at 100 rpm at 37°C. Ten ml aliquots were collected during exponential (EX) growth (OD_600nm_ = 0.4) for the SNM cultures and during EX (OD_600nm_ = 0.4) and early stationary phase (ST) for the BHI cultures. One volume of RNAprotect® (Qiagen; Hilden, Germany) was added to halt transcription and to stabilise the RNA by subsequent incubation at room temperature for 30 min. Bacterial cells were subsequently harvested by centrifugation at 6,000 × *g* for 10 min at room temperature and the RNA extracted from the cell pellets as described below.

### *In vivo* sampling, ethics, consent and permissions

To investigate the suitability of the pan-genome approach for evaluating transcripts derived from *in vivo* samples (where strain/genome sequence information may be unavailable), swabs from the anterior nares were collected from a healthy volunteer where informed consent has been obtained for the analyses described (approved by the Ethical Committee of the Medical Faculty of the University of Münster and of the Ärztekammer Westfalen-Lippe file number 2010-468-f-S). Persistent colonization had been verified by inoculation of cotton swabs of the anterior nares on selective chromogenic media (CHROMagar^TM^ *S*. *aureus*) on four different timepoints during a one year period and incubation for 48 h at 30°C in ambient air. Suspicious colonies were confirmed as *S*. *aureus* by amplification of the *nuc* gene as previously described [18]. Characterization of the colonizing strain was performed by Multilocus Sequence Typing (MLST) as previously described [19] and the sequence type assigned as ST30 using the MLST database (http://saureus.mlst.net).

The sample for metatranscriptomic analysis was taken using a standard swabbing technique with FLOQSwabs™ (COPAN; Brescia, Italy), placed in RNase free centrifuge tubes (Eppendorf; Hamburg, Germany) and 80 μl of RNAprotect™ Bacteria Reagent (Qiagen) added directly to the surface of the swab. Samples were subsequently kept for 2 min at room temperature and the RNA extracted from whole swabs as described below.

### RNA processing

For RNA extraction, stabilised cell pellets were re-suspended in 700 μl of cold RLT buffer (Qiagen) supplemented with 1% β-mercaptoethanol (Sigma; Taufkirchen, Germany) and transferred to lysing matrix B tubes (MP Biomedicals; Heidelberg, Germany) placed on ice. In case of *in vivo* samples, whole swabs were added directly to the lysing matrix tubes comprising the same quantity (700 μl) of the RLT/β-mercaptoethanol solution. Samples were disrupted using a FastPrep-24® instrument (MP Biomedicals) at an intensity of 5.5 for 40 s, incubated on ice for 4 min and subjected to a second disruption as described above. Cell debris was removed by centrifugation at 13,500 × *g* for 3 min at 4°C and the supernatant transferred to a 1.5 ml RNase free Biopur™ centrifuge tube (Eppendorf) and the RNA extracted using the RNeasy® Mini Kit according to the manufacturer’s instructions, including optional DNase treatment on the column (Qiagen). RNA was eluted with nuclease-free water (Ambion; Darmstadt, Germany) and concentrated by ethanol precipitation using standard procedures. RNA extracts prepared from nasal swabs were pooled yielding a single *in vivo* anterior nares sample.

To remove any possibly contaminating DNA, a further DNase treatment step was performed on the *in vitro* samples using the Turbo DNA-free™ kit (Invitrogen; Darmstadt, Germany). Ribosomal RNA was subsequently depleted from all samples using Terminator™ 5′-Phosphate-Dependent Exonuclease (Epicentre; Hessisch Oldendorf, Germany) according to the manufacturer’s instructions. Samples were concentrated by ethanol precipitation, re-diluted in nuclease-free water and the RNA integrity and quantity measured using the Agilent 2100 Bioanalyzer (Agilent Technologies; Santa Clara, CA, USA) and NanoDrop 1000 spectrophotometer (Thermo Scientific; Waltham, MA, USA).

Enriched mRNA fractions (500 ng) were amplified using the MessageAmp™ II-Bacteria RNA amplification kit (Ambion) according to the manufacturer’s instructions although with a T7 primer modified to include a *Bpm*I restriction site for removal of poly-A tails prior to sequencing, as described elsewhere [20]. Amplified samples were subsequently converted to double-stranded (ds-) cDNA using the SuperScript® Double-Stranded cDNA Synthesis Kit (Invitrogen), purified using the GeneClean® Turbo for PCR Kit (MP Biomedicals), and endonuclease digestion performed using *Bpm*I according to the manufacturer’s recommendations (NEB; Frankfurt am Main, Germany). Samples were further purified using the same GeneClean® kit and the processed ds-cDNA used in the preparation of Illumina RNAseq libraries as described below.

### Illumina library construction and sequencing

A total of 7 libraries (4 *in vitro* samples of USA300 strain LAC and IPL32 cells collected during EX and ST growth phases in BHI media, 2 *in vitro* samples of USA300 and IPL32 collected during EX growth phase in SNM and 1 *in vivo* human anterior nares sample from a documented *S*. *aureus* carrier) were prepared for sequencing using the Paired-End DNA Sample Preparation kit (Illumina; San Diego, CA, USA) using 1 μg of ds-cDNA according to the manufacturer’s instructions and were assessed using the Agilent 2100 Bioanalyzer (Agilent Technologies). Libraries prepared from *in vitro* samples were sequenced on the Illumina® Genome Analyzer GAIIx platform using the Paired-End Cluster Generation Kit v5 (Illumina), with libraries multiplexed on a single lane (9 pM/library) and sequenced to 150 cycles in both directions. To maximise the sequence lengths which could be used for more definitive assignment, the *in vivo* library was sequenced on a single lane of the Illumina® MiSeq platform according to the manufacturer’s instructions (10 pM/library), generating 150 base paired-end reads. Image analysis and base calling of all sequence reads were performed using the Illumina pipeline version 1.8.

### RNAseq bioinformatics pipeline and data analysis

All raw sequence reads generated from the transcriptomic libraries were filtered and subsequently trimmed using fastx_trimmer available in the FASTX-Toolkit (version 0.0.13.2) [http://hannonlab.cshl.edu/fastx_toolkit/] if the quality value fell below Q ≤ 15, if homopolymers were ≥ 8 nt; and there were sequence ambiguities. Then, only those sequences that were a minimum of 80 nt were retained in the dataset. These filtered reads were subsequently collapsed into representative reads (RR) using the fasta_collapser script within the same suite.

Collapsed RR datasets were subsequently processed to remove contaminating human and/or ribosomal RNA sequences. Human associated RNAs were identified and removed from the *in vivo* sample using BLAT searches [21] of an in-house database of the human genome repository (human RefSeq, chromosome records with gap adjusted concatenated NT_contigs) downloaded from the NCBI ftp site, blastdb. Sequences with ≥70% identity in the query were removed from the dataset. Ribosomal sequences contained within the RR datasets were detected with HMMER (version 3.0) [22] using models based on multiple sequence alignments of the 5S, 16S and 23S rRNAs [23], and blastn searches of an in-house database containing ribosomal proteins from *S*. *aureus* (as constructed from the 25 genomes used in establishing the pan-genome).

The remaining sequence reads from the *in vivo* sample were treated as putative prokaryotic mRNAs from which the *S*. *aureus* associated fraction were retrieved using the same blast-based strategy implemented *in silico* using as cut-off an alignment score of 80% when aligned a database of nucleotide sequences from 25 *S*. *aureus* genomes (see above for details). These reads, along with those generated from the *in vitro* samples, were subsequently mapped against the OG database (representing the *S*. *aureus* pan-genome) using rpstblastn. An alignment score of ≥ 60% was used for the positive assignment of sequences to the orthologous groups. Reads below this cut-off were blasted against the exclusive protein sequences using blastx.

To aid in elucidating the effectiveness of the OG database for assigning reads from *in vivo* samples or divergent strains lacking a genome sequence, reads from all samples were also compared to a single reference genome, *S*. *aureus* USA300 TCH1516 via blastn. To aid in elucidating the effectiveness of the OG database for assigning reads from *in vivo* samples or divergent strains lacking a genome sequence, reads from all samples were also compared to a single reference genome, *S*. *aureus* USA300 TCH1516 via blastn with an alignment score of ≥ 60% as cut-off value.

To compare gene expression under different *in vitro* conditions, all read counts were resampled (999 times) up to 340,000 reads using an in-house perl script. To compare in vivo and in vitro expression, the numbers of reads assigned by each approach were rank-ordered (GEO series accession number GSA56294) and the degree of bias introduced by the two algorithms (ppstblast versus blastn) compared using cluster analysis (group average) in PRIMER-E [12].

Comparisons of the global transcriptomic profiles from the 7 RNAseq libraries were performed by Principal Coordinate Analysis (PCoA) and agglomerative hierachical clustering, with the Bray-curtis similarity algorithm used to assess the similarity between samples in PRIMER-E [12]. To visualise the global differences in expression between the 7 conditions, the relative abundances of read counts were plotted using the OG database as a reference using CIRCOS [24].

### Availability of supporting data

Sequences generated from the transcriptomic libraries were deposited to the NCBI Gene Expression Omnibus (GEO) repository under the accession number GSA56294.

Position specific scoring matrices (PSSMs), the preformated RPS Blast Database, strain specific proteins, and the names and identification numbers for all the proteins used in the construction of the OG database can be downloaded from http://dx.doi.org/10.7910/DVN/JRPDWL

## Results and Discussion

### Compilation of the *S*. *aureus* “pan-genome” and genomic features

In order to discern the number of *S*. *aureus* genome sequences required to define its pan-genome and thus to create a suitable reference database from which RNAseq reads generated from divergent strains and *in vivo* samples can reliably be interrogated, publicly-available completed genomes and the respective encoded proteins (32 as of January 2013) were obtained from the National Center for Biotechnology Information (NCBI) ftp site. These included strains of different clonal complex (CC) lineages and niches (see S1 Table). Protein sequences were assigned to orthologous groups (OGs) [8,9] in order to create a list of OGs belonging to either the core, accessory or unique components of the pan-genome. Core OGs comprised, among others *met*E genes encoding 5-methyltetrahydropteroyltriglutamate-homocysteine methyltransferases involved in methionine symthesis (OG 1412, comprising USA300HOU_0377) or *sbn*A genes encoding the staphylobactin biosynthesis protein SbnA (OG 166, comprising USA300HOU_0127) (see S2 Table).

**Fig 1:**
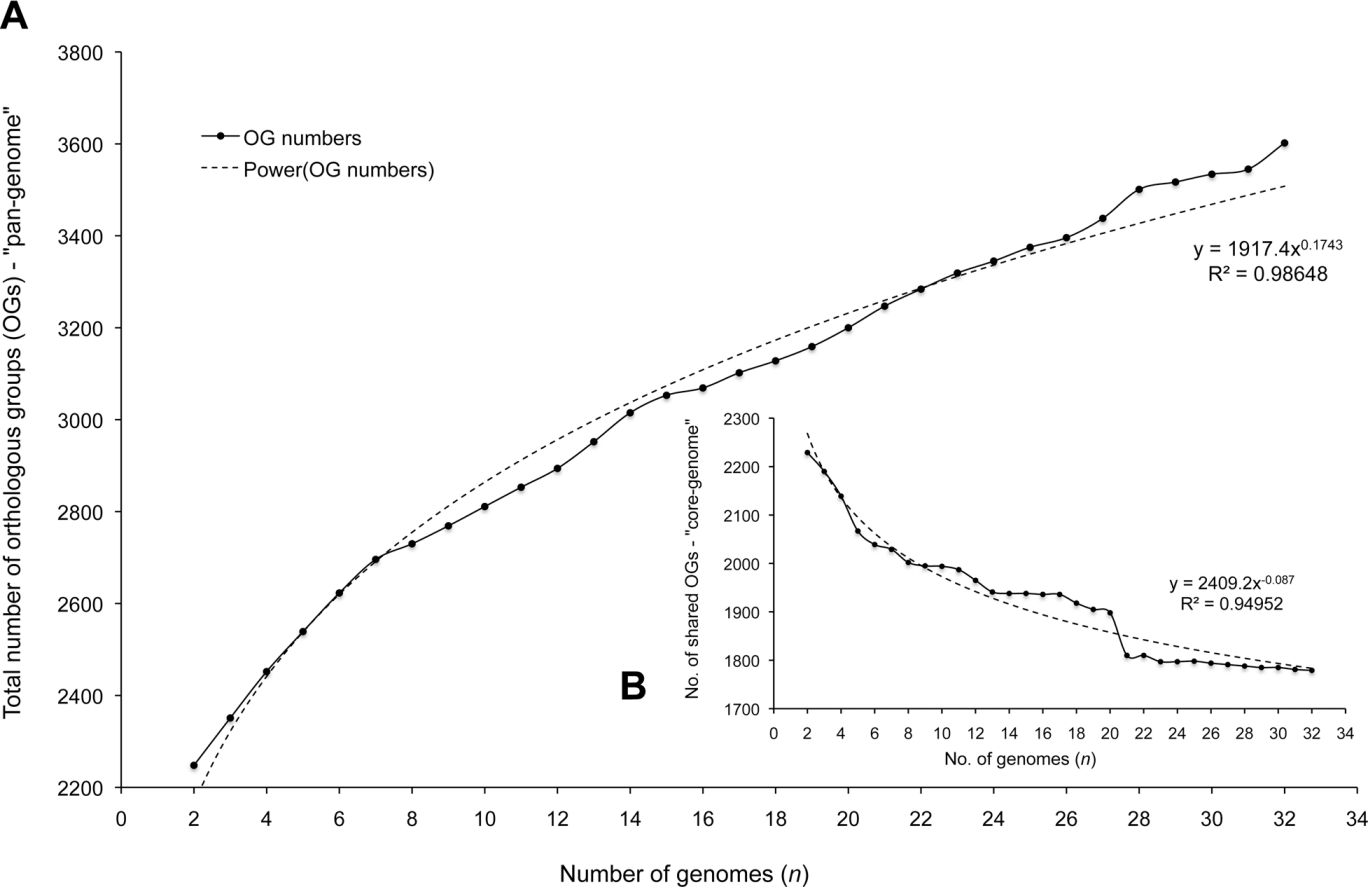
*S*. *aureus* pan- and core-genome. Saturation plots depicting the pan- and core-genome sizes of *S*. *aureus* as estimated by plotting as a function of the number of genomes (*n*), the accumulation of (A) the total number of unique orthologous groups (OGs) and (B) the number of shared OGs. A total of 32 genomes were evaluated with the accumulation of OG numbers (solid lines) also fit by power curves (broken lines) and the respective equations and R^2^ values inset.

Plotting the number of OGs encoded by core and accessory genes as a function of the number of sequentially included genomes revealed that the number of “new” OGs inherently increased by increasing the number of genomes (Fig 1). However, this plot tended toward a finite number of “new” identified genes and fitted a power law curve (R^2^ = 0.986), thus indicating a closed pan-genome [4,8,9]. It also indicated that the majority of the *S*. *aureus* pan-genome content can be captured in < 21 genomes. Plotting the number of only core OGs as a function of the number of genomes revealed that the number of core OGs decayed exponentially with increasing genome numbers, reaching a plateau at approximately 1,800 OGs (Fig 1).

Twenty-five genomes were selected (S1 Fig) and deemed as being sufficient for cataloging the majority of core and accessory genes in the pan-genome of *S*. *aureus*. From these 25 genomes, a total of 65,557 protein sequences (2,622 ± 98 CDS per genome) were clustered into 4,198 OGs, of which 1,813 (corresponding to 45,589 proteins and 69.5% of the total protein sequences considered) belonged to the core and 1,653 (corresponding to 19,236 proteins and 29.3% of the total protein sequences considered) to the accessory component of the pan-genome. The remaining 732 proteins genes (1.1% of the total protein sequences considered) were encoded by strain-specific genes (see S3 Table).

Previous pan-genome comparative analysis among different pathogens (including 17 strains of *S*. *aureus*) [8] reported a significantly lower number of OGs (3,155). Similar numbers could be infered from a study which inluded 16 *S*. *aureus* genomes and identified a total of 2,861 OGs [9]. Different methodologies to identify orthologous genes, typically using the reciprocal best hit and different clustering methods with different stringencies have been applied [25]. Compared to other methods [8,25], the OrthoMCL routine used here provides a more robust method that enables separating diverged paralogs and distant orthologs that could be incorrectly assigned by the reciprocal best hit method [11]. However, independent of the method used, the number of core OGs in *S*. *aureus* can be assumed to be lower than 2,000, whereas the numbers of auxiliary functions can be strongly influenced by the clustering method used.

Global comparisons of the OG content amongst the 25 *S*. *aureus* strains used in the construction of the pan-genome database revealed that all strains exhibited a reasonably high level of similarity (88–99.8%) with an average similarity of approximately 90% observed across all strains (S1 Fig). The different strains clustered according to their respective clonal complex (CC), thus reflecting a common clonal ancestry as reported elsewhere [26–28].

An inclusion in the pangenome of two genomes representing CC22 and CC45 (*S*. *aureus* HO 5096 0412, NC_017763 and *S*. aureus CA-347, PRJNA197422, respectively), also identified as colonizers of human anterior nares [29,30] increased the amount of OGs by only 22 corresponding to 0.5%. This is in accordance with above assumption that the majority of *S*. *aureus* genes are covered by the OG database created. However, as inherent to such databases, they cannot cover all genes present in the diversity of *S*. *aureus* strains.

### Using the “pan-genome” approach for assessing the *S*. *aureus* transcriptome

The OG database was then used to analyze the transcriptome of *S*. *aureus* under seven conditions of differing complexities and challenges. The experimental sets comprised the well characterized and widely used strain of methicillin-resistant *S*. *aureus* (MRSA) USA300 strain LAC grown in Brain Heart Infusion (BHI) media sampled at exponential and stationary growth phases, the methicillin-resistant *S*. *aureus* strain IPL32 [16] grown in BHI media sampled at exponential and stationary growth phases, both strains grown in a Synthetic Nasal Medium (SNM) that mimicks human nasal secretions [17] sampled at exponential growth phase and a *S*. *aureus* ST30 residing in the anterior nares of a documented *S*. *aureus* carrier. In total, between 2–9 million reads were obtained from the seven RNAseq libraries of which 0.5–3.5 million reads comprised bacterial mRNA that was used for further analyses (Table 1).

**Table 1:** Bioinformatic filtering of the 7 transcriptomic (RNAseq) libraries.

	USA300 (BHI EX)	USA300 (BHI ST)	IPL32 (BHI EX)	IPL32 (BHI ST)	USA300 (SNM EX)	IPL32 (SNM EX)	Unknown strain (*in vivo*)
# reads generated per sample	4,556,926	3,859,002	2,002,756	2,996,033	2,699,959	4,793,450	8,710,509
# reads after quality filtering	3,835,671	3,280,598	1,705,070	2,520,263	2,352,219	4,145,532	8,149,262
# mRNA reads after removal of ribosomal content	3,502,961	2,580,884	1,241,790	2,001,662	1,827,741	2,882,451	574,668
# reads assigned to OGs (incl. exclusive genes)	404,437	615,878	342,556	586,745	909,945	1,506,207	1,009
# OGs (incl. exclusive genes) detected (of 4198)	2,864	2,866	2,781	2,771	2,936	2,969	436
# reads assigned to USA300_TCH1516 genome	324,238	577,559	308,625	556,057	894,261	1,460,466	941
# genes detected (of USA300_TCH1516 2608 genes)	2,446	2,436	2,292	2,308	2,474	2,383	400
% increase in reads when using OG assignment	20	6	9	5	2	3	7

To determine the best strategy for analyzing the relative gene expression, the presumptive *S*. *aureus* mRNA reads from the *in vitro* conditions were subsequently blasted against both the OG database generated in this study and against a representative reference genome (USA300_TCH1516) using the rpstblastn and blastn algorithms respectively. Agglomerative hierarchical cluster analysis (group average) revealed that, independent of the blast algorithm implemented, the samples grouped according to the condition, thus negating the likelihood of any computationally introduced bias (S2 Fig). An increased amount of mRNA reads (3% - 20%) could be assigned to *S*. *aureus* using the OG database (Table 1).

Thus, an advantage of using the OG database over using a reference genome for anaylsis of RNAseq transcription data is the high coverage provided by the pan-genome of *S*. *aureus* as the OG database allowed the identification of a number of genes in IPL32, which were absent from the genome of *S*. *aureus* USA300_TCH1516. As an example, the gene encoding the staphylococcal collagen-binding protein Cna (*cna*, ST398NM01_2740), an important virulence factor of *S*. *aureus* [31], was found highly expressed by *S*. *aureus* IPL32 in all growing conditions only when the bacterial transcriptome was analysed using the OG database. Also the gene encoding the staphylococcal enterotoxin L (*sel*, SAV2008) was found to be expressed by IPL32 under any of the three *in vitro* conditions analyzed only when the transcriptome analysis was perfomed using the OG database. In case of USA300 strain LAC, the greater amount of sequencing reads mapped using the OG database can be explained by an incomplete annotation of the USA300 strain TCH1516 reference genome.

### Discriminating reads originating from *S*. *aureus* from those originating from *S*. *epidermidis*

The mapping of reads from complex community metatranscriptome samples to specific species can be specialy challenging when the samples are derived from a niche co-habited by closely related strains. This can be the case in metatranscriptomes obtained from the human anterior nares where *S*. *aureus* shares the niche with the closely related *S*. *epidermidis* [6], known to encode proteins with a high level of similarity to those of *S*. *aureus* [9,32,33]. Thus, the level to which reads specific to *S*. *aureus* could reliably be assigned was determined using an *in silico* mixed bacterial community. For this purpose, different proportions of randomly extracted reads from the genomes of *S*. *aureus* (6850), *S*. *epidermidis* (RP62A) and *Dolosigranulum pigrum* (ATCC 51524, like *Staphylococcus* sp. a member of the class Bacilli), were combined into three different *in silico* generated metatranscriptomes and the sequencing reads were interrogated at two levels of stringency (see Materials and Methods) against the nucleotide sequence database comprising the complete genomes from the 25 *S*. *aureus* strains used here and against a database consisting of the sequences of the *S*. *epidermidis* strains ATCC 12228 and RP62A. Importantly, at stringency levels of 70% and 80%, respectively, only < 0.5% or < 0.2% reads originating from *D*. *pigrum* could not clearly be assigned. In contrast, at a stringency level of 70%, approximately 15–23% of reads originating from either *S*. *aureus* or *S*. *epidermidis* could not be properly assigned to the respective species, due to the high similarity of gene sequences (S3 Fig). At a stringency level of 80%, only 8–16% of reads could not be properly assigned. This stringency level was considered therefore the most appropriate for assigning reads from the *in vivo* metatranscriptome, as it could cover all reads originating from *S*. *aureus* with only a small level of misannotation.

### Assessing the *S*. *aureus* transcriptome under *in vitro* and *in vivo* conditions

From the metatranscriptome obtained from the nasal community of a *S*. *aureus* carrier, a total of 1,333 reads could be defined as being *S*. *aureus* specific reads after blasting against the nucleotide sequence database comprising the complete genomes from the 25 *S*. *aureus* strains used here. Out of these, 1,009 reads were mapped to the OG database, where the remaining reads were identified as signal recognition particle (SRP) or tmRNA among others. Only 941 reads were mapped to *S*. *aureus* after blasting against the *S*. *aureus* USA300 reference genome. Clearly, the OG database allows identifying proteins that may have not been annotated in a selected reference genome. It also easily allows deducing the identity of the protein a sequence read is matching to, as the encoded proteins are sorted into annotated OGs. This is in clear advantage compared to the best hit results when blasting against a complete sequence database, which may not indicate the nature of the encoded protein.

**Fig 2:**
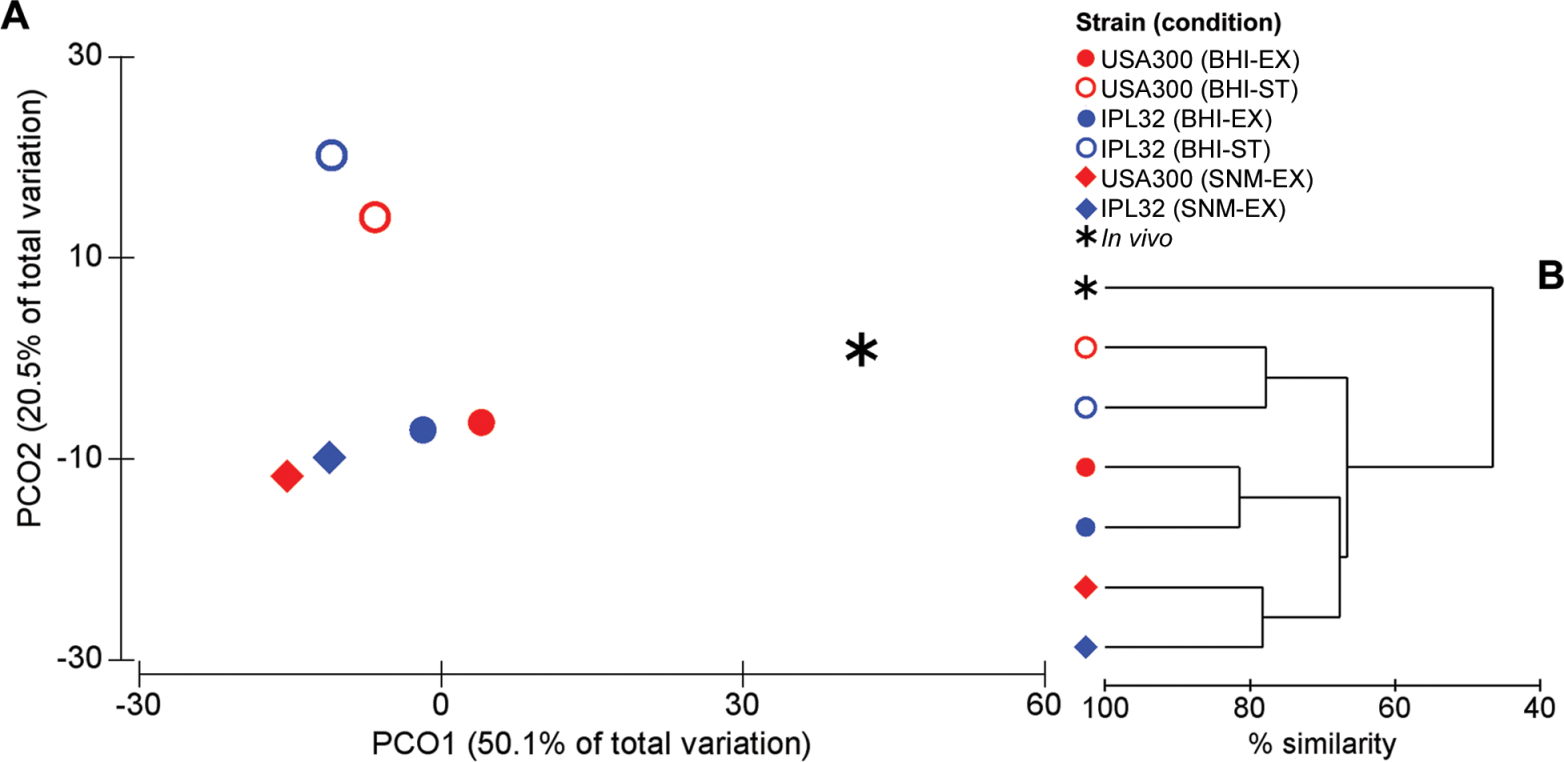
Comparison of the global *S*. *aureus* transcriptome under *in vivo* and *in vitro* growth conditions. Comparison of the global *S*. *aureus* transcriptomic profile from 7 RNAseq libraries using (A) Principal Coordinate Analysis (PCoA) and (B) agglomerative hierachical clustering (group-average linkage). Conditions included: *in vitro* exponential and stationary phase cultures of *S*. *aureus* USA300 strain LAC grown in Brain Heart Infusion (BHI) media; *in vitro* EX and ST phase cultures of *S*. *aureus* strain IPL32 grown in BHI; *in vitro* EX phase cultures of both strains grown in a Synthetic Nasal Medium (SNM) that mimics the composition of the human nasal secretions [17]; and *in vivo S*. *aureus* within the complex bacterial community of the human anterior nares. The Bray-curtis similarity algorithm was used to assess the similarity between samples based on the relative abundance of transcripts for each OG, where 70.6% of the total variation could be explained by PCO1 and PCO2.

A data matrix comparing the assigned reads counted against the OG database (S2 Table) was subsequently compiled for each of the 7 conditions and the different conditions were compared using group-average agglomerative hierarchical clustering and Principal Co-ordinate Analysis (PcoA) (Fig 2). The global transcription profiles of the strains USA300 LAC and IPL32 grown under identical *in vitro* conditions always clustered tightly together, sharing roughly 80% similarity within each of the 3 *in vitro* growth conditions. Furthermore, all transcriptomes from the 6 *in vitro* samples, irrespective of strain, media or growth phase differences shared a global similarity of ≥ 67%. However, the transcriptome of *S*. *aureus* colonizing the human anterior nares shared on average only 47% similarity with any of the transcriptomes from *S*. *aureus* growing under different *in vitro* conditions. This highlights the strong influence of the environmental conditions encountered within the host in the adaptive transcriptional response of *S*. *aureus*.

**Fig 3:**
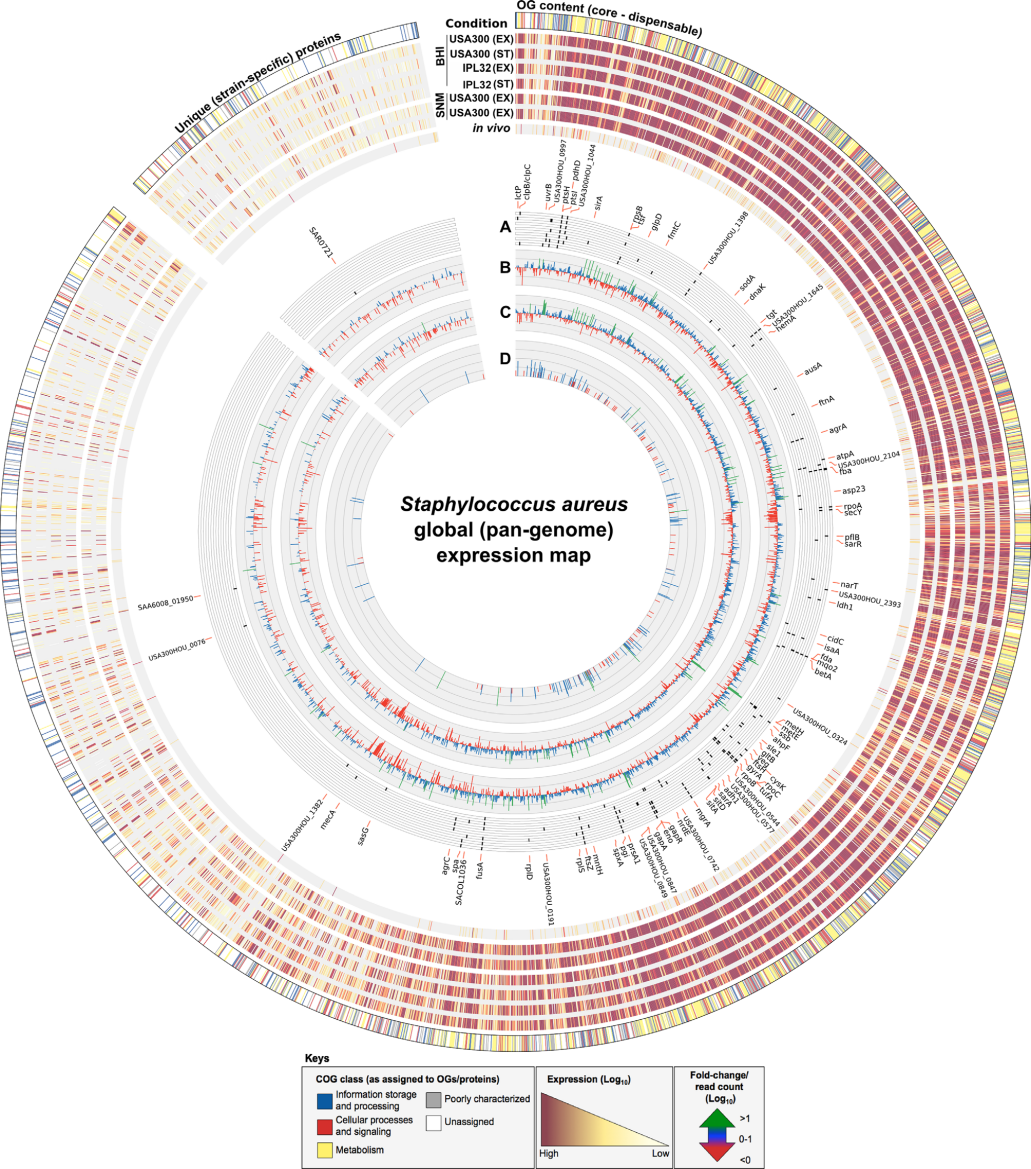
Global (pan-genome) expression map of *in vitro* and *in vivo* derived *S*. *aureus* transcriptomes. Circular ideogram depicting variations in gene expression between *S*. *aureus* strains, *in vitro* growth media and *in vivo* conditions as mapped according to the 3466 core/variable OGs and 732 unique (strain-specific) proteins defined for the *S*. *aureus* pan-genome. RNAseq generated reads (plotted as log_10_ expression values) were assigned to their respective OGs/proteins by rpstblastn (ordered from core–variable–unique) with each OG defined according to its major Clusters of Orthologous Groups (COG) class (outer circle). Expression values from a total of 7 conditions were mapped and represent (from outer to the inner): *S*. *aureus* USA300 *in vitro* exponential (EX) and stationary (ST) phase growth in Brain Heart Infusion (BHI) media; *S*. *aureus* IPL32 *in vitro* EX and ST phase growth in BHI; *S*. *aureus* USA300 and IPL32 *in vitro* EX phase growth in Synthetic Nasal Medium (SNM); and transcripts taken from an *in vivo* (metatranscriptomic) sample generated from the human anterior nares of an *S*. *aureus* carrier. Inner circles represent: (A) the top 25-ranked most highly expressed genes in each of the 7 conditions (based on abundance of transcripts) and plotted as a tile graph where black lines (or tiles) correspond to a highly expressed gene under a given condition (ordered according to the outer circles), with those specific to *in vivo* conditions marked in bold; (B) fold-change (log_10_) of *in vitro* EX growth of USA300 in SNM versus BHI media; (C) fold-change (log_10_) of *in vitro* EX growth of IPL32 in SNM versus BHI media; and (D) total *S*. *aureus-*specific read counts (log_10_) from the *in vivo* human anterior nares condition. Keys denote the color scheme used to distinguish COG classes and expression and fold-change/read count values.

The differences in gene expression between *S*. *aureus* USA300 strain LAC growing in SNM versus complex medium have recently been analysed by microarray and quantitative RT-PCR [17]. Although the differences in gene expression reported in that study could be confirmed here, the RNAseq method used in this study exhibited an increased sensitivity when compared with microarray analysis [34]. The major differences between the transcriptome of cells growing exponentially in complex medium versus those growing exponentially in SNM were due to the limiting availability of iron in SNM (see Fig 3). According to the OG database, genes of the *isd*BACDEF gene cluster (SACOL1138, USA300HOU_1064–1068) as well as *isd*H (USA300HOU_1720) encoding iron regulated surface determinants were upregulated by *S*. *aureus* up to 5-fold in SNM. The *sbn*ABCDEFGHI operon (USA300HOU_0127–0135), which encodes proteins for the biosynthesis of the staphyloferrin B siderophore [35], was upregulated by *S*. *aureus* in SNM up to 10–200 fold. An only approximately 2-fold change upregulated gene expression was determined in these genes by microarray analysis, contrasting the 40-fold upregulation determined by quantitative RT-PCR [17]. Other genes related to iron homeostasis found higly induced by *S*. *aureus* in SNM were those encoding staphyloferrin A synthesis (*sfa*CBAD, USA300HOU_2170–2173, upregulated 2–30 fold) [36] as well as the respective transport systems (*sir*ABC, USA300HOU_0126–0124, upregulated 2–9 fold) and *hts*ABC (USA300HOU_2169–2167, upregulated 2–4 fold) [37,38]. Further evidence for the need to acquire iron in SNM is provided by the high abundance of transcripts of genes encoding α-hemolysin (*hly*, USA300HOU_1099, upregulated 19 fold) or phenol soluble modulins β (USA300HOU_1112–1113, upregulated 74–90 fold). These are released by *S*. *aureus* to both kill the host immune cells and thereby evading the host immune defenses [39] as well as to break down red blood cells with the concomitant release of hemoglobin, which can be used by *S*. *aureus* as a source of iron [40].

Also, evident by the transcriptome analysis using the OG database was the central role of the amino acid metabolism for *S*. *aureus* growing in SNM as shown by the immense increase in transcripts of genes encoding amino acid transporters and enzymes involved in amino acid biosynthesis. These observations are in accordance with the previously described transcriptional response of *S*. *aureus* in SNM using a microarray [17]. In particular, genes encoding methionine synthesis proteins were transcribed at a higher level by *S*. *aureus* in SNM than in complex medium (e.g. *met*ICFE, USA300HOU_0380–0377 upregulated 29-104-fold). Among these genes, cytathionine-c-synthase MetI has been shown to be crucial for growth of *S*. *aureus* in SNM, where methionine was absent [17]. The transcription of other amino acid biosynthetic cluster such as *his*EFAHBCDGZ (USA300HOU_2673–2681) was also found to be significantly increased in *S*. *aureus* growing in SNM (2-42-fold).

Although the set of genes differentially expressed by the nasal isolate *S*. *aureus* IPL32 when growing in complex medium versus SNM was highly similar to that observed in *S*. *aureus* USA300 strain LAC, some differences were also observed, which may reflect the better adaptation of IPL32 to the nasal environment. As an example, the genes encoding enzymes involved in purine metabolism (*pur*EKCSQLFMHD, USA300HOU_1009–1018) were up-regulated up to 50-fold by strain IPL32, but only up to 4-fold by USA300 strain LAC. Purine metabolism is important for DNA synthesis as well as for energy production and it has been shown to be essential for *S*. *aureus* to survive within the host [41]. All *cap*ABCEFHIKLMNOP genes (USA300HOU_0163–0178) were found to be expressed to a high extent in strain IPL32 and were upregulated in SNM compared to complex medium (up to 26 fold for *cap*M USA300HOU_0175). This would be in agreement with a recent report proposing capsule expression as one of the mechanism used by *S*. *aureus* to persist in the anterior nares by hindering phagocytocis [42]. Accordingly, *S*. *aureus* recovered from the nares of experimentally colonized mice expressed high levels of capsule, and the ability of a capsule-defective mutant to persist in the nares was reduced in comparison to that of the parent strain [43]. A two- to three-fold higher expression of capsule biosynthesis genes has also been reported for *S*. *aureus* USA300 strain LAC cultured in SNM compared to complex medium [17], however, recent analysis indicated the presence of 4 conserved mutations in the *cap* locus of USA300 strains where a promoter mutation attenuates expression and mutations in *capD* and *capE* ablate production or activity of key enzymes [44]. In accordance expression of *cap* genes by *S*. *aureus* USA300 strain LAC was poor compared to that of strain IPL32.

An advantage of the OG database was that it enabled the identification of genes which were upregulated by IPL32 during growth in SNM, such as *seb* encoding staphylococcal enterotoxin B (SACOL0907, 6-fold upregulated by IPL32 in SNM), and which were absent from USA300 strain TCH1516 and absent from strain LAC or not expressed by the latter. Other proteins which were absent in USA300 strain TCH1516 and absent from strain LAC or not expressed under any of the applied conditions by the latter but upregulated by IPL32 in SNM comprised a putative exported protein similar to the SAS03071 protein of *S*. *aureus* MSSA476 (upregulated 8 fold) and a putative membrane protein (similar to SAS0369, upregulated 5 fold).

The OG database also constitutes an useful tool to assign transcriptional reads from metatranscriptome samples originating from bacterial complex communities to specific species. The suitability of the method was demonstrated in this study using a metatranscriptome originating from the nasal community of a *S*. *aureus* human carrier. Although only a relatively low number of sequence reads could be affiliated to *S*. *aureus*, these reads allowed to get a snapshot of the most highly expressed genes by this strain in its human niche.

Such reads may identify essential survival pathways required by *S*. *aureus* to persist in the nasal cavity, which may then be used as potential targets for the development of novel decolonization strategies. As an example, the *sas*G gene (SACOL2505) which did not rank under the 1000 most highly *S*. *aureus* expressed genes under any of the *in vitro* conditions analyzed, was among the top 10 expressed genes in the nasal cavity (see S4 Table) and a detailed analysis of the sequence reads confirmed, that they originated from *S*. *aureus*. Actually, the ability of *S*. *aureus* cells to adhere to human nasal epithelial cells is imperative for colonization and the *sas*G gene product was shown to promote a strong adhesion to squamous cells [45–48]. This seems to be mediated by the production of fibrils that completely cover the complete bacterial-cell surface and probably physically mask the expression of smaller adhesins [46].

Overall, the level of stringency used for a Blastn search against *Staphylococcus* genome databases allowed the successful separation of transcripts originating from different species and the OG database allowed a rapid identification of *S*. *aureus* genes transcribed *in vivo*.

## Conclusions

The use of an RNAseq approach coupled with a catalogue of orthologous genes obtained from the *S*. *aureus* pan-genome allowed a reliable and rapid assessment of transcripts, particularly when considering samples that contain distinct clones or bacterial species closely related to *S*. *aureus*. Environment-specific and strain-specific responses could be identified as well as genes highly expressed by *S*. *aureus in vivo* in the human host. The next step will be to use this approach to achieve a deeper understanding of the *S*. *aureus* trancriptional response and of its interaction with other bacteria within the host (*e*.*g*. anterior nares or skin). This will provide highly valuable information regarding the colonization, adaptation and persistence of *S*. *aureus* as a colonizer of the human host that can eventually lead to the development of more efficient decolonization strategies.

## Supporting Information

S1 Fig Hierarchical clustering of *S*. *aureus* strains. Dendrogram constructed by agglomerative hierarchical clustering (group-average linkage) based on a protein relative abundance matrix (compiled from the numbers of proteins per OG per genome) generated from comparisons between 25 *S*. *aureus* strains. The percentage similarity between strains was calculated using the Bray-curtis similarity algorithm. Strains are colour-coded by their clonal complex (CC) type and their specific association with animal (AA) and/or human (HA) hosts.
(TIF)

S2 Fig Cluster plot comparing rpstblastn and blastn algorithms. Dendrogram constructed by agglomerative hierachical clustering (group-average) based on a relative abundance matrix constructed from comparisons between the rpstblastn and blastn interrogation of reads (against the OG database and the *S*. *aureus* USA300_TCH1516 reference genome) obtained from RNAseq libraries generated from *in vitro* cultures of *S*. *aureus* strains USA300 and IPL32. The strain, growth media (Brain Heart Infusion—BHI and Synthetic Nasal Medium—SNM) and growth phases (exponential—EX and stationary—ST) are indicated in the key. The percentage similarity between conditions was caluclated using the Bray-curtis similarity algorithm.
(TIF)

S3 Fig I*n silico* analysis of randomly selected *S*. *aureus*, *S*. *epidermidis* and *D*. *pigrum* transcriptomic reads Evaluation of blast searches from a batch of 6000 randomly selected reads (of 100 nt in length) compiled in A) equal quantities B) a 1:9:0 ratio and C) a 7:2:1 ratio from the genomes of *S*. *aureus* 6850, *S*. *epidermidis* RP62A and *D*. *pigrum* ATCC 51524, as interrogated against a database comprising nucleotide sequences from 25 *S*. *aureus* genomes (S1 Table) and a database of 2 *S*. *epidermidis* strains (ATCC 12228 and RP62A). The diagram shows the expected number of reads (first column) with reads originating from *S*. *aureus* given in read, those originating from *S*. *epidermidis* in blue and those from *D*. *pigrum* given in green. The second and third column show the number of reads assigned to *S*. *aureus* and *S*. *epidermidis*, respectively, when the genome databases were interrogated using an alignment score of > 70% (alignment length × % identity/query length) as cut-off and the fourth and fifth column the number of reads when the genome databases were interrogated using an alignment score of 80% as cut-off.
(TIF)

S1 Table Complete list of *S*. *aureus* genomic sequences used in comparative evaluations. (XLSX)

S2 Table Transcripts abundances of genes expressed by *S*. *aureus* during each of the 7 conditions. The list comprises abundances in *in vitro* exponential (EX) and stationary phase (ST) cultures of *S*. *aureus* USA300 strain LAC grown in Brain Heart Infusion (BHI) media; *in vitro* EX and ST phase cultures of *S*. *aureus* strain IPL32 grown in BHI; *in vitro* EX phase cultures of both strains grown in a Synthetic Nasal Medium (SNM) replicating human nasal secretions, and *in vivo S*. *aureus*-specific component of the anterior nares. Each OG is indicated by a representative gene and the gene name (if available). OGs where the number of reads assigned to this OG were above 0.005 of the total number of reads under one of the seven conditions are shown.
(XLSX)

S3 Table Clusters of Orthologous Groups of proteins (COGs) distribution between strains used in the *S*. *aureus* pan-genome construction. COGs were assigned to 51930 (from a total of 65557) unique protein sequences retrieved from the National Center for Biotechnology Information (NCBI) ftp site for the genomes of the 25 *S*. *aureus* strains by blastp queries against the myva database [49] using a cut-off of ≥ 50% coverage and identity. ^a^The total numbers of COGs (and the corresponding number of protein sequences) are reported for the major COG classes and associated categories. ^b^Variation in COG abundance across the 25 genomes was estimated using Pielou's evenness [50] where (in plotting the inverse values): 0 = maximally even, with the same number of protein sequences occurring in a given COG across all 25 genomes; and 1 = maximally uneven, with variable numbers of protein sequences assigned to each COG across the 25 genomes. ^c^Percentages of the total number of variable COGs assigned to each of the major COG classes and the percentage numbers of those occurring above an evenness threshold value of ≥ 0.01 per category, class and as a fraction of total number of COGs assigned to all strains (i.e. 1653). *Total number includes those protein sequences (i.e. 4621) which could be assigned to more than one COG.
(DOCX)

S4 Table Summary list of the most highly expressed OGs across the 7 conditions. This list comprises the top 25-ranked OGs within each of the 7 conditions; *in vitro* exponential (EX) and stationary phase (ST) cultures of *S*. *aureus* USA300 strain LAC grown in Brain Heart Infusion (BHI) media; *in vitro* EX and ST phase cultures of *S*. *aureus* strain IPL32 grown in BHI; *in vitro* EX phase cultures of both strains grown in a Synthetic Nasal Medium (SNM) replicating human nasal secretions, and *in vivo S*. *aureus-*specific component of the anterior nares. Each OG is indicated by a representative gene, and the COG code, COG category and description are given.
(XLSX)
