# Supplementary figures and images for: Application of a Novel “Pan-Genome”-Based Strategy for Assigning RNAseq Transcript Reads to Staphylococcus aureus Strains
Source: PLoS One. 2015 Dec 30;10(12):e0145861. doi: 10.1371/journal.pone.0145861 (PMC4696825; doi:10.1371/journal.pone.0145861)

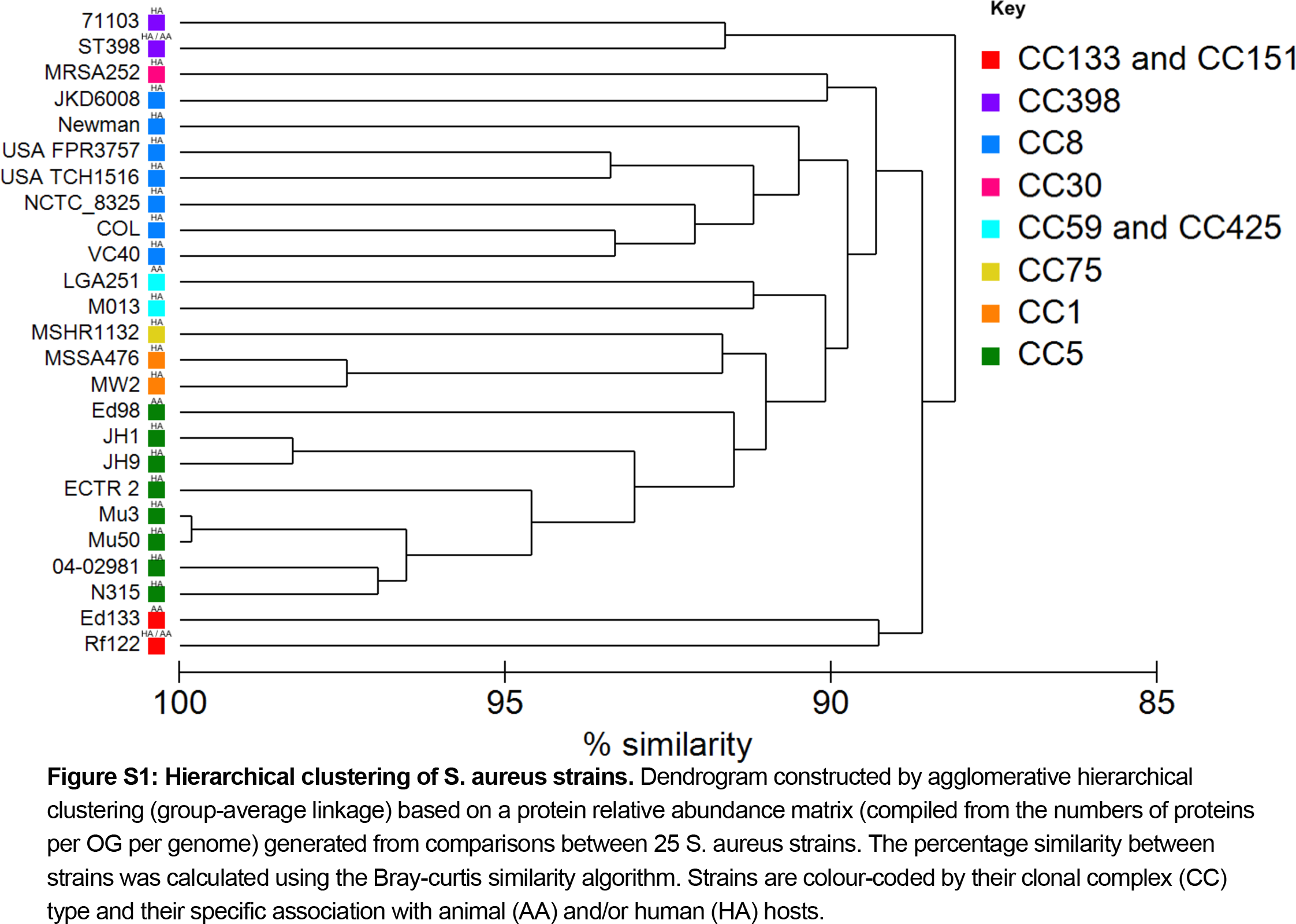

Supplement: S1 Fig — Dendrogram constructed by agglomerative hierarchical clustering (group-average linkage) based on a protein relative abundance matrix (compiled from the numbers of proteins per OG per genome) generated from comparisons between 25 S. aureus strains. The percentage similarity between strains was calculated using the Bray-curtis similarity algorithm. Strains are colour-coded by their clonal complex (CC) type and their specific association with animal (AA) and/or human (HA) hosts. (TIF) [file pone.0145861.s001.tif]

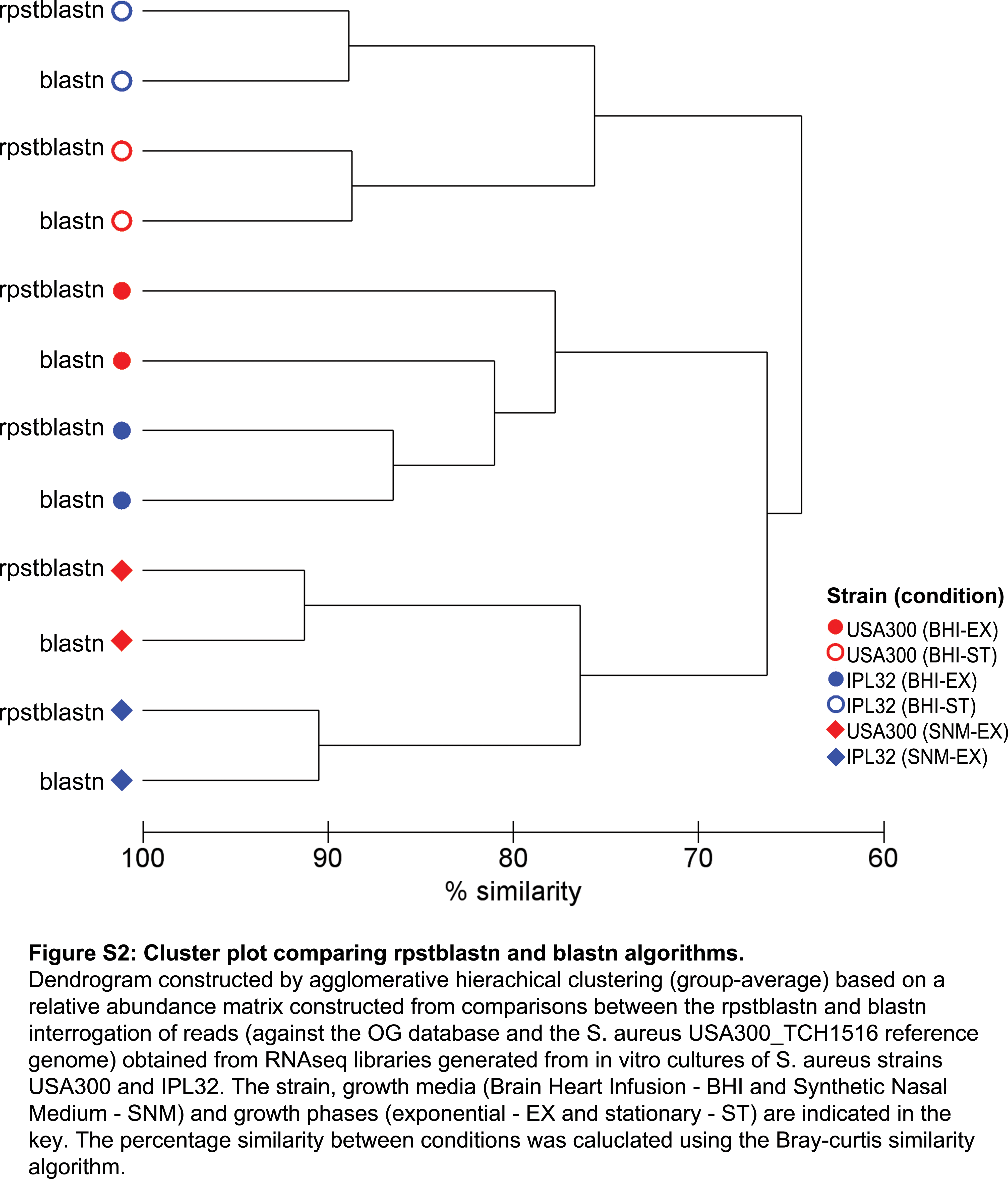

Supplement: S2 Fig — Dendrogram constructed by agglomerative hierachical clustering (group-average) based on a relative abundance matrix constructed from comparisons between the rpstblastn and blastn interrogation of reads (against the OG database and the S. aureus USA300_TCH1516 reference genome) obtained from RNAseq libraries generated from in vitro cultures of S. aureus strains USA300 and IPL32. The strain, growth media (Brain Heart Infusion—BHI and Synthetic Nasal Medium—SNM) and growth phases (exponential—EX and stationary—ST) are indicated in the key. The percentage similarity between conditions was caluclated using the Bray-curtis similarity algorithm. (TIF) [file pone.0145861.s002.tif]

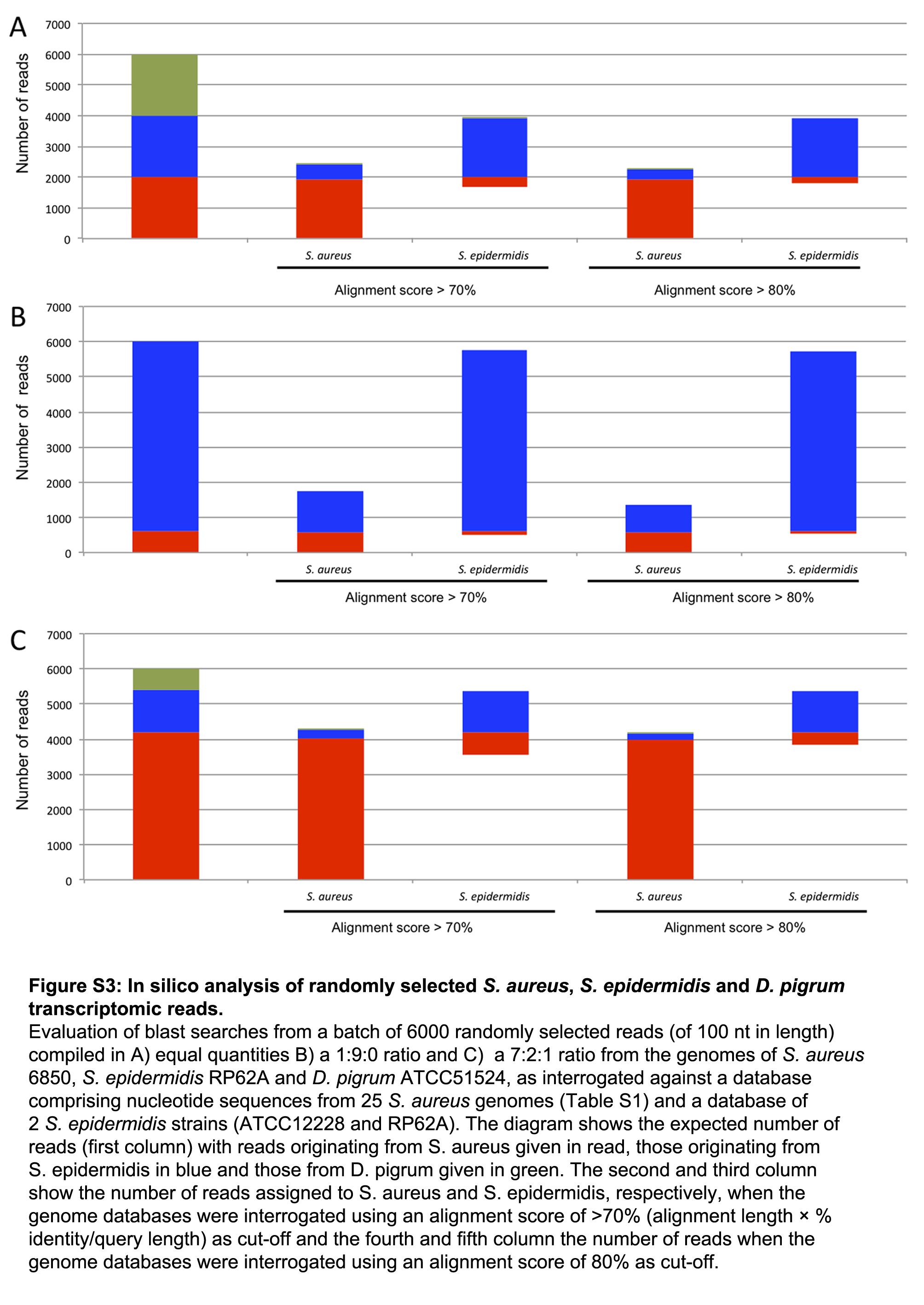

Supplement: S3 Fig — The diagram shows the expected number of reads (first column) with reads originating from S. aureus given in read, those originating from S. epidermidis in blue and those from D. pigrum given in green. The second and third column show the number of reads assigned to S. aureus and S. epidermidis, respectively, when the genome databases were interrogated using an alignment score of > 70% (alignment length × % identity/query length) as cut-off and the fourth and fifth column the number of reads when the genome databases were interrogated using an alignment score of 80% as cut-off. (TIF) [file pone.0145861.s003.tif]
