# Supplementary material for: Application of a Novel “Pan-Genome”-Based Strategy for Assigning RNAseq Transcript Reads to Staphylococcus aureus Strains
Source: PLoS One. 2015 Dec 30;10(12):e0145861. doi: 10.1371/journal.pone.0145861 (PMC4696825; doi:10.1371/journal.pone.0145861)
Supplement: S3 Table — COGs were assigned to 51930 (from a total of 65557) unique protein sequences retrieved from the National Center for Biotechnology Information (NCBI) ftp site for the genomes of the 25 S. aureus strains by blastp queries against the myva database [49] using a cut-off of ≥ 50% coverage and identity. aThe total numbers of COGs (and the corresponding number of protein sequences) are reported for the major COG classes and associated categories. bVariation in COG abundance across the 25 genomes was estimated using Pielou's evenness [50] where (in plotting the inverse values): 0 = maximally even, with the same number of protein sequences occurring in a given COG across all 25 genomes; and 1 = maximally uneven, with variable numbers of protein sequences assigned to each COG across the 25 genomes. cPercentages of the total number of variable COGs assigned to each of the major COG classes and the percentage numbers of those occurring above an evenness threshold value of ≥ 0.01 per category, class and as a fraction of total number of COGs assigned to all strains (i.e. 1653). *Total number includes those protein sequences (i.e. 4621) which could be assigned to more than one COG. (DOCX) [file pone.0145861.s006.docx]

## Supplementary Table 3. Clusters of Orthologous Groups of proteins (COGs) distribution between strains used in the *S. aureus* pan-genome construction

| **COG major class^a^** | **COG category code** | **COG category name** | **No. COGs** | **No. variable COGs (>0.01)^b^** | **Evenness range^b^** | **% variable COGs with threshold value >0.01^c^** | | | **Total % variable COGs per class** |
| --- | --- | --- | --- | --- | --- | --- | --- | --- | --- |
|  |  |  |  |  |  | **per category** | **per class** | **per total** |  |
| Information Storage and Processing  343 (11094) | B | Chromatin structure and dynamics | 2 | 0 | 0.0000 | 0 | 0 | 0 | 25.1 |
|  | J | Translation, ribosomal structure and biogenesis | 135 | 13(6) | 0.0188-0.0549 | 4.4 | 1.7 | 0.4 |  |
|  | A | RNA processing and modification | 1 | 0 | 0.0000 | 0 | 0 | 0 |  |
|  | K | Transcription | 88 | 32(14) | 0.0005-0.0476 | 15.9 | 4.1 | 0.8 |  |
|  | L | Replication, recombination and repair | 117 | 41(29) | 0.0011-0.0641 | 24.8 | 8.5 | 1.8 |  |
| Cellular Processes and Signaling  258 (9317) | D | Cell cycle control, cell division, chromosome partitioning | 17 | 6(1) | 0.0006-0.0106 | 5.9 | 0.4 | 0.1 | 30.0 |
|  | V | Defense mechanisms | 24 | 12(5) | 0.0008-0.0235 | 20.8 | 1.9 | 0.3 |  |
|  | T | Signal transduction mechanisms | 41 | 13(6) | 0.0006-0.1350 | 14.6 | 2.3 | 0.4 |  |
|  | M | Cell wall/membrane/envelope biogenesis | 77 | 22(6) | 0.0006-0.0234 | 7.8 | 2.3 | 0.4 |  |
|  | N | Cell motility | 9 | 2(2) | 0.0266-0.1350 | 22.2 | 0.8 | 0.1 |  |
|  | U | Intracellular trafficking, secretion, and vesicular transport | 29 | 5(4) | 0.0018-0.0260 | 13.8 | 1.6 | 0.2 |  |
|  | O | Post-translational modification, protein turnover, and chaperones | 61 | 7(3) | 0.0004-0.0175 | 4.9 | 1.2 | 0.2 |  |
| Metabolism  620 (23169) | C | Energy production and conversion | 83 | 14(3) | 0.0003-0.0212 | 3.6 | 0.5 | 0.2 | 21.2 |
|  | G | Carbohydrate transport and metabolism | 93 | 23(1) | 0.0004-0.0202 | 1.1 | 0.2 | 0.1 |  |
|  | E | Amino acid transport and metabolism | 143 | 34(7) | 0.0004-0.0397 | 4.9 | 1.1 | 0.4 |  |
|  | F | Nucleotide transport and metabolism | 59 | 6(1) | 0.0008-0.0130 | 1.7 | 0.2 | 0.1 |  |
|  | H | Coenzyme transport and metabolism | 83 | 11(3) | 0.0004-0.0231 | 3.6 | 0.5 | 0.2 |  |
|  | I | Lipid transport and metabolism | 44 | 11(2) | 0.0006-0.0185 | 4.5 | 0.3 | 0.1 |  |
|  | P | Inorganic ion transport and metabolism | 94 | 30(5) | 0.0002-0.0329 | 5.3 | 0.8 | 0.3 |  |
|  | Q | Secondary metabolites biosynthesis, transport, and catabolism | 21 | 3(1) | 0.0006-0.0245 | 4.8 | 0.2 | 0.1 |  |
| Poorly Characterized  432 (12971) | R | General function prediction only | 201 | 53(18) | 0.0003-0.0788 | 9.0 | 4.2 | 1.1 | 23.6 |
|  | S | Function unknown | 231 | 49(23) | 0.0008-0.0445 | 10.0 | 5.3 | 1.4 |  |
| **TOTAL:** 1653 COGs (56551 proteins*) | | | | | | | | | |

COGs were assigned to 51930 (from a total of 65557) unique protein sequences retrieved from the National Center for Biotechnology Information (NCBI) ftp site for the genomes of the 25 *S. aureus* strains by blastp queries against the myva database [49] using a cut-off of > 50% coverage and identity. ^a^The total numbers of COGs (and the corresponding number of protein sequences) are reported for the major COG classes and associated categories. ^b^Variation in COG abundance across the 25 genomes was estimated using Pielou's evenness [50] where (in plotting the inverse values): 0 = maximally even, with the same number of protein sequences occurring in a given COG across all 25 genomes; and 1 = maximally uneven, with variable numbers of protein sequences assigned to each COG across the 25 genomes. ^c^Percentages of the total number of variable COGs assigned to each of the major COG classes and the percentage numbers of those occurring above an evenness threshold value of > 0.01 per category, class and as a fraction of total number of COGs assigned to all strains (i.e. 1653). *Total number includes those protein sequences (i.e. 4621) which could be assigned to more than one COG.
